# Engineering Cold Stress Tolerance in Crop Plants

**DOI:** 10.2174/138920211794520178

**Published:** 2011-03

**Authors:** Gulzar S Sanghera, Shabir H Wani, Wasim Hussain, N.B Singh

**Affiliations:** 1 Shere Kashmir University of Agricultural Sciences and Technology of Kashmir, Rice Research and Regional Station, Khudwani, Anantnag, 192102, Kashmir, India; 2 Central Institute of Temperate Horticulture, Srinagar, Kashmir, India; 3 Department of Plant Breeding and Genetics, COA, Central Agricultural University, Imphal, Manipur, 795 004, India

**Keywords:** Cold stress, genetic engineering, transcription factors, crop plants.

## Abstract

Plants respond with changes in their pattern of gene expression and protein products when exposed to low temperatures. Thus ability to adapt has an impact on the distribution and survival of the plant, and on crop yields. Many species of tropical or subtropical origin are injured or killed by non-freezing low temperatures, and exhibit various symptoms of chilling injury such as chlorosis, necrosis, or growth retardation. In contrast, chilling tolerant species are able to grow at such cold temperatures. Conventional breeding methods have met with limited success in improving the cold tolerance of important crop plants involving inter-specific or inter-generic hybridization. Recent studies involving full genome profiling/ sequencing, mutational and transgenic plant analyses, have provided a deep insight of the complex transcriptional mechanism that operates under cold stress. The alterations in expression of genes in response to cold temperatures are followed by increases in the levels of hundreds of metabolites, some of which are known to have protective effects against the damaging effects of cold stress. Various low temperature inducible genes have been isolated from plants. Most appear to be involved in tolerance to cold stress and the expression of some of them is regulated by C-repeat binding factor/ dehydration-responsive element binding (*CBF/DREB*1) transcription factors. Numerous physiological and molecular changes occur during cold acclimation which reveals that the cold resistance is more complex than perceived and involves more than one pathway. The findings summarized in this review have shown potential practical applications for breeding cold tolerance in crop and horticultural plants suitable to temperate geographical locations.

## INTRODUCTION

Abiotic stresses adversely affect growth, productivity and trigger a series of morphological, physiological, biochemical and molecular changes in plants. Cold stress is a major environmental factor that limits the agricultural productivity of plants in hilly areas. Plants respond and adapt to this stress to survive under stress conditions at the molecular and cellular levels as well as at the physiological and biochemical levels. However, expression of a variety of genes is induced by different stresses in diverse plants.

Low temperature often affects plant growth and crop productivity, which causes significant crop losses [1]. Plants differ in their tolerance to chilling (0-15 ºC) and freezing (< 0ºC) temperatures. In general, plants from temperate climatic regions are considered to be chilling tolerant with variable degree, and can increase their freezing tolerance by being exposed to chilling, non-freezing temperatures, a process known as cold acclimation [2], which is associated with biochemical and physiological changes [3-5] and ultimately showed marked changes in gene expression, biomembrane lipid composition, and small molecule accumulation [6]. Besides, plants of tropical and subtropical origins. are sensitive to chilling stress and lack the mechanism of cold acclimation. Low temperature resistance in plants is a very complex trait, involving many different metabolic pathways and cell compartments [7]. Conventional breeding methods have met with limited success in improving the cold tolerance of important crop plants involving inter-specific or inter-generic hybridization. Besides, *in vitro* induced variations have also been applied to improve the abiotic stress tolerance of various crop plants but without much success. The conventional breeding approaches are limited by the complexity of stress tolerance traits, low genetic variance of yield components under stress condition and lack of efficient selection criteria. It is important, therefore, to look for alternative strategies to develop cold stress tolerant crops.

**Fig. (1):**
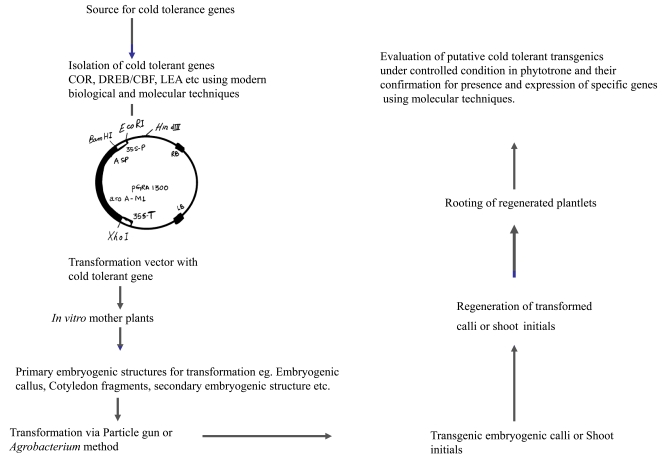
Scheme for genetic engineering of crop plants against cold stress.

Biotechnology offers new strategies that can be used to develop transgenic crop plants with improved tolerance to cold stress. Rapid advance in recombinant DNA technology and development of precise and efficient gene transfer protocols have resulted in efficient transformation and generation of transgenic lines in a number of crop species [8-10] (Fig. **1**). A number of genes have been isolated and characterized that are responsive to freezing stress. Many studies have suggested that cold regulated gene expression is critical in plants for both chilling tolerance [11] and cold acclimation [12, 13]. Advent of molecular tools has made it possible to select directly at the gene label without waiting for the phenotype to show up. Transgenic approach is being pursued actively throughout the world to improve traits including tolerance to biotic and abiotic stresses in a number of crops [14]. As compared to other stresses, plant responses to cold stress are complex, so the prospects of improving cold tolerance in crops seem not to be very bright. Despite this, efforts have been made during the last two decades to generate transgenic lines of different crops, which have shown improved tolerance to cold stress. Therefore, it is important to use most appropriate tools that help in reaching the goals. The genotype designed should be better than the available ones and must reach the farmers. An attempt has been made in this article to review the various mechanisms and genes involved in cold acclimatization and the possibilities where transgenic technology has been explored for breeding cold tolerance in crop plants.

## MORPHO-PHYSIOLOGICAL BASIS OF COLD TOLERANCE

A large number of studies have evaluated different plant species tolerant to different stresses such as drought, salinity and cold. However, less detail is given with regard to the methods used to evaluate the stress response; these studies may bring about some misleading conclusions from an agronomic or physiology perspective [15]. This is particularly important, in order to closely mimic the life span of most crops under cycles of stress, rather than short exposure to very severe stresses; although we agree that short exposures to stress are certainly adequate if the purpose is to assess gene expression only. In this section, we focus on the agronomic/physiological perspective and do not mean to challenge the quality of the work done to assess gene expression. Our intention is to try to reconcile both approaches (agronomic and molecular) toward a common focus: breeding cold tolerance. Though precise details about the protocols used to evaluate the performance of plants to any given stress are very essential to assess the performance of materials.

The temperate and cool regions are those where altitudes ranged from 1600-2500 m amsl (above mean sea level) and temperature during crop growth period ranged from 5-20 ^0^C [16]. In temperate regions, low temperature is the primary abiotic stress which limits the crop productivity. The low temperature at seedling and reproductive stages is the major problem, results in slow establishment and low seed set which leads to poor yield of the crop [15]. The low temperature limits the crop productivity when temperatures remain above freezing that is > 0 ^o^C, it is called as chilling stress. Chilling sensitive cultivars are typically tropical genotypes. There is wide range of cold stress in temperate areas differing in both timing and intensity of low temperature. Yield losses are more severe when cold stress occurs during reproductive stage/ anthesis in rice which lead to high spikelet sterility [17]. Ability of crop genotypes / lines to survive / perform better under low temperature than other genotypes is called as cold tolerance. Ordinarily, it is the consequence of cold hardening that is an earlier exposure to a low temperature for a specific period as a result of which chilling tolerance of the concerned plants increases. Cold tolerance involves increased chlorophyll accumulation, reduced sensitivity of photosynthesis, improved germination, pollen fertility and seed set which are desirable as:

## INCREASED CHLOROPHYLL ACCUMULATION

Low temperature inhibits chlorophyll accumulations in actively growing leaves. In rice, cold tolerant lines, for example, *japonica* accumulates more chlorophyll under cold stress than do cold sensitive line, for example of *indica* rice [18]. Rasolofo [19] evaluated 181 accessions to identify donor and outstanding cold tolerant lines using leaf discolouration score (1-3) and found 19 remained green (dark) after 10 days in the 12 ^o^C cold water tank. Sanghera *et al*. [17] found 18 cold tolerance IRCTN rice genotypes based on dark green colour and high spikelet fertility (>90%) under temperate conditions.

## REDUCED SENSITIVITY OF PHOTOSYNTHESIS

Chloroplast and photosynthesis is major site of cold injury. Tolerance in these aspects is expressed in native vegetation adapted to growing under cool conditions. The reduced sensitivity of photosynthesis to cold has been observed in maize inbreds adapted to low temperature which is partly related to specific enzymes of the process [20].

## IMPROVED GERMINATION

Genetic variation in cold tolerance at germination and seedling stage has been documented. Saini and Tandon [21] found that L62G, Heng Jodo, Jodo, Heugdo, IRAT 102, Khonorullo, K 78-28, Daegaldo, Mujudo and L62-2A genotypes were cold tolerant having more than 85% germination and good seedling vigour (score 3) at an average of 11 ^o^C field temperature

### Improved Pollen Fertility and Seed Set 

Cold tolerance at reproductive stage is expressed as improved seed set and pollen fertility. It is largely a function of floral structure and function under stress. Lia *et al*. [22] reported plant cold tolerance in rice is associated with anther size, number of pollen grain, diameter of fertile pollen grains at booting stage. However, Sanghera *et al*. [17] reported that cold tolerance is associated with high spikelet fertility (>90%) and well panicle exsertion under temperate conditions.

Cold snaps cause a reaction in the plant that prevents sugar getting to the pollen. Without sugar there is no starch build-up which provides energy for pollen germination. And without pollen, pollination cannot occur, thereby, no grain is produced. CSIRO has found that all the ingredients for starch are present but they are not getting into the pollen grain where they are needed. A cell layer surrounding the pollen, called the ‘tapetum’, is responsible for feeding the pollen with sugar. The tapetum is only active for 1-2 days – so if a cold snap occurs at this time, then there is no further chance for pollen growth. But the sugar cannot freely move into the tapetum and pass through it to the pollen. Instead the sugar has to be broken down then transported in bits to the pollen. ‘Invertase’ is the catalyst that helps in breakdown of the sugar molecule to transport it into the tapetum before it is transported to the pollen [23]. Quantities of invertase are decreased in conventional rice when it is exposed to cold temperatures, but they remain at normal levels in a cold tolerant variety when it experiences cold. By comparing a cold tolerant strain of rice with conventional rice, CSIRO has found that the gene responsible for invertase looks exactly the same in the cold tolerant variety as it does in conventional rice. So the invertase gene itself does not make the rice plant cold tolerant – but instead a mechanism that regulates the invertase gene is different. Early research indicates that the invertase gene is regulated by the hormone abscisic acid (ABA). Oliver *et al*. [24] has experimented with injecting plants with ABA – the resulting rice plants are sterile, just like that if they experienced a cold snap. Also, ABA levels increase when conventional rice is exposed to cold, but they remain the same in the cold tolerant variety. Recent studies have indicated that the difference between cold-sensitive and tolerant rice is due to a different ability to control ABA levels [25]. It has also been shown that this mechanism may require interactions with other plant hormones like auxins [26]. Further, Zhao *et al*. [25] also reported that low temperature turns off the genes responsible for sugar transport into the pollen grains and therefore starch cannot be produced in the pollen in cold conditions. Cold did not cause repression of sugar delivery in cold tolerant Chinese rice and fertile pollen was still produced following cold treatment. The sugar metabolism genes also continued to function normally during cold treatment of cold tolerant rice. Ample genetic variation for cold tolerance is available in well adapted breeding population. Germplasm collected from high altitude and low temperature areas, cold tolerant mutant, somaclonal variants and wild species can be exploited for breeding improved cold tolerant genotypes in hilly areas [15].

## MECHANISMS FOR UNDERSTANDING TOLERANCE TO COLD INJURY

Low temperature has a huge impact on the survival and geographical distribution of plants. It affects a range of cellular metabolisms in plant system depending on the intensity and duration of the stress. When exposed to low temperatures, plants respond with changes in their pattern of gene expression and protein products. Different studies have indicated that the membrane systems of the cell are the primary site of freezing injury in plants [2, 27]. In addition, it is well established that freeze-induced membrane damage results primarily from the severe dehydration associated with freezing [27, 28]. Many species of tropical or subtropical origin are injured or killed by nonfreezing low temperatures, and exhibit various symptoms of chilling injury such as chlorosis, necrosis, or growth retardation. In contrast, chilling-tolerant species are able to grow at such cold temperatures.

However, multiple forms of membrane damage can occur as a consequence of freeze induced cellular dehydration including expansion-induced-lysis, lamellar-to-hexagonal-II phase transitions, and fracture jump lesions [28]. Thus, a key function of cold acclimation should be to stabilize membranes against freezing injury. Indeed, cold acclimation prevents expansion-induced-lyses and the formation of hexagonal II phase lipids in rye and other plants [28]. This ability to adapt has an impact on the distribution and survival of the plant, and on crop yields. Multiple mechanisms appear to be involved in this stabilization. The best documented are changes in lipid composition [28]. Secondly, temperature induced change in membrane fluidity is another consequences in plants during temperature stresses and might represent a potential site of perception and/or injury [29, 30]. Adaptation of living cells to chilling temperatures is a function of alteration in the membrane lipid composition by increased fatty acid unsaturation. Genetically engineered tobacco plants over-expressing chloroplast glycerol-3-phosphate acyltransferase (GPAT) gene (involved in phosphatidyl glycerol fatty acid desaturation) from squash (*Cucurbita maxima*) and *A. thaliana* showed an increase in the number of unsaturated fatty acids and a corresponding decrease in the chilling sensitivity. At low temperature, greater membrane lipid unsaturation appears to be crucial for optimum membrane function. An *Arabidopsis* fatty acid biosynthesis (*FAB1*) mutant with more saturated membranes showed decreased quantum efficiency of photosystem II (PSII), chlorophyll content and the amount of chloroplast glycerolipids after prolonged exposure to low temperature [31]. A triple mutant fatty acid desaturation (*fad3-2 fad7-2 fad8*) devoid of trienoic fatty acids (18:3 or 16:3) produced a phenotype similar to *FAB1*, when plants were subjected to prolonged low temperature exposure [32]. Similarly, f*ad5* and *fad6* mutants with more saturated membranes became chlorotic and showed growth retardation during low temperature incubation [33]. In addition to membrane unsaturation, it appears that lipid asymmetry in the membrane also contributes to membrane physical structure at low temperature [34]. The accumulation of sucrose and other simple sugars that typically occurs with cold acclimation also seems likely to contribute to the stabilization of membranes as these molecules can protect membranes against freeze-induced damage *in vitro* [35, 36]. 

Besides, protective chaperone like function of LEA proteins acting against cellular damage has been proposed, indicating the role of LEA proteins in anti aggregation of enzymes under desiccation and freezing stresses. There is emerging evidence that certain novel hydrophilic and late embryogenesis abundant (LEA) polypeptides also participate in the stabilization of membranes against freeze-induced injury. These hydrophilic and late embryogenesis abundant polypeptides are predicted to contain regions capable of forming amphipathic α-helices which are shown to have strong effect on intrinsic curvature of monolayers and their propensity to form hexagonal II phase. They are said to defer their formation at lower temperatures [37]. There is another evidence that freeze-induced production of reactive oxygen species contributes to membrane damage and that intercellular ice can form adhesions with cell walls and membranes and cause cell rupture [38]. Further, there is evidence that protein denaturation occurs in plants at low temperature [39] which could potentially result in cellular damage. In these cases, the enhancement of antioxidative mechanisms [40], increased levels of sugars in the apoplastic space [41], and the induction of genes encoding molecular chaperones [39], respectively, could have protective effects.

In cold stress-tolerant plants, many genes involved in the synthesis of osmoprotectants—organic compounds such as amino acids (e.g. proline), quaternary and other amines (e.g. glycinebetaine and polyamines) and a variety of sugars and sugar alcohols (e.g. mannitol, trehalose and galactinol) that accumulate during osmotic adjustment—have been used. Both cold-stress-induced transcripts and constitutively expressed transcripts need to be processed, exported to the cytoplasm and kept in conformations that are competent for translation. RNA can fold into extensive secondary structures that could interfere with its function, and cold temperatures exacerbate this interference. Many genes that respond to multiple stresses like dehydration and low temperature at the transcriptional level are also induced which protects the cell from dehydration and chilling. In order to restore the cellular function and make plants more tolerant to stress, transferring a single gene encoding a single specific stress protein may not be sufficient to reach the required tolerance levels. To overcome such constraints, enhancing tolerance towards multiple stresses by a gene encoding a stress inducible transcription factor that regulates a number of other genes is a promising approach. In bacteria, nucleic-acid-binding cold shock proteins (CSPs) accumulate at cold temperatures and function as transcription antiterminators or translational enhancers by destabilizing RNA secondary structure [42]. Some CSP-domain-containing proteins in plants are upregulated by cold stress, and might function as RNA chaperones in the regulation of translation [43, 44]. A different cold-responsive nucleic-acid-binding protein, a zincfinger- containing glycine-rich RNA-binding protein from *Arabidopsis* designated *atRZ-1a*, is also upregulated by cold stress, and genetic analysis supports its function in freezing tolerance [45]. Compared to other organisms, plants have the largest number of DEAD-box RNA helicase genes [46]. One of these helicases, which is encoded by the *Arabidopsis* low expression of osmotically responsive genes4 (*LOS4*) gene, is essential for plant tolerance of chilling and freezing stress [47]. *LOS4 *is required for efficient export of RNA from the nucleus to the cytoplasm [48]. The *Arabidopsis* nucleoporin *AtNUP160* suppressor of auxin resistance1 (*SAR1*) also controls RNA export, and is crucial for chilling and freezing tolerance [49]. Both *LOS4* and *AtNUP160* proteins are enriched at the nuclear rim [47, 49]. Defects in the nucleocytoplasmic transport of RNA seem to affect cold tolerance preferentially, because the *LOS4* and *AtNUP160* mutant plants do not have severe growth or developmental phenotypes, nor are they strongly altered in the tolerance of other abiotic stresses.

## KEY PLAYERS INVOLVED IN COLD RESPONSIVE PATHWAYS

Cold tolerance is the result of complex physiological mechanisms involving many cell and plant traits. Earlier studies have shown that the genetic control of cold tolerance is complex and can be regarded as polygenic [50] and the mechanism of how these genes controlled cold tolerance is still not fully clear. Therefore, the foundation for a better molecular and genetic understanding of the cold responsive pathways will improve our knowledge and pave the way for the development of improved methodologies for cold tolerance screening. Key to the tolerance of plants to abiotic stresses is a complex network of transcription factors and other regulatory genes that control multiple defense enzymes, proteins and pathways [51]. The discovery of gene expression change during cold acclimation was the starting of exploration of antifreezing molecular mechanisms. In this context, Zhao *et al*. [25] reported that gene expression profiling using DNA chips indicated that large numbers of genes were differentially expressed under cold stress. They identified 242 unique genes that are expressed differentially between cold sensitive and cold tolerant rice. These genes are involved in processes such as senescence, cell death, male sterility and plant hormone response. Similarly, global transcript profiling analyses indicated that > 10% of genes in the *Arabidopsis* genome were regulated during cold acclimation [52-55]. Transcriptome analysis using microarray technology is a powerful technique, which has proven very useful for discovering many stress-inducible genes involved in stress response and tolerance [25, 55-57]. Genes involved in stress signal sensing and a cascade of stress-signaling in *A. thaliana* has been of recent research interest. Components of the same signal transduction pathway may also be shared by various stress factors such as drought, salt and cold. Although there are multiple pathways of signal-transduction systems operating at the cellular level for gene regulation.

In past, it has been reported that genes induced during stress conditions function not only in protecting cells from stress by producing important metabolic proteins, but also in regulating genes for signal transduction in the stress response [52]. Several stress induced cor genes such as rd29A, cor15A, kin1 and cor6.6 are triggered in response to cold treatment, ABA and water deficit stress in the early stages of the osmotic stress response.

Several stress induced cor genes such as rd29A, cor15A, kin1 and cor6.6 are triggered in response to cold treatment, ABA and water deficit stress in the early stages of the osmotic stress response. Similarly, a cis-acting element, dehydration responsive element (DRE) identified in *A.* *thaliana*, is also involved in ABA-independent gene expression under drought, low temperature and high salt stress conditions in many dehydration responsive genes like rd29A that are responsible for dehydration and cold-induced gene expression. Thus, clearly, the overexpression of some drought-responsive transcription factors can lead to the expression of downstream genes and the enhancement of abiotic stress tolerance in plants. Thus, these gene products are classified into two groups [53, 54]. The first group of proteins that probably function in stress tolerance includes chaperones, LEA proteins, osmotin, antifreeze proteins, mRNA-binding proteins, some key enzymes for osmolyte biosynthesis (like proline, water channel proteins, sugar and proline transporters, detoxification enzymes), enzymes for fatty acid metabolism (proteinase inhibitors, ferritin) and lipid-transfer proteins [58]. It has been reported that some of these stress-inducible genes specially encoding proteins (such as enzymes for osmolyte biosynthesis, LEA proteins and detoxification enzymes) have been overexpressed in transgenic plants and produce stress-tolerant phenotypes in the transgenic plants [51, 56]. These results indicate that the gene products of the stress-inducible genes really function in stress tolerance. The second group contains protein factors involved in regulation of signal transduction and gene expression that probably function during stress response [59]. This group includes various transcription factors that regulate different stress-inducible genes collectively or separately, and may constitute gene networks. Seki *et al*. [59] reported that some of these regulatory pathways are also involved in drought-, cold-, or high-salinity stress responses. Though, the clear cut functions of most of these genes are not fully understood. Functional analysis of these stress-inducible transcription factors will provide precise information on the complex regulatory gene networks that are involved in responses to drought, cold, and high-salinity stresses [60, 25]. Some of these stress-inducible regulatory genes that encode proteins (transcription factors) have been overexpressed in transgenic plants and generate stress-tolerant phenotypes in them [61, 62].

## COLD TOLERANCE USING TRANSGENIC APPROACHES

When a plant is subjected to abiotic stress, a number of genes are turned on, resulting in increased levels of several metabolites and proteins, some of which may be responsible for conferring a certain degree of protection to these stresses. A key to progress towards breeding better crops under stress has been to understand the changes in cellular, biochemical and molecular machinery that occur in response to stress. A key to progress towards breeding better crops under stress has been to understand the changes in cellular, biochemical and molecular machinery that occur in response to stress which in turn provides new tools and strategies to improve the environmental stress tolerance of crops. Since freezing tolerance is a multigenic trait [63], transformation of a single functional gene appears to have a limited effect on crop freezing tolerance [64]. Because many aspects of cold adaptation process are under transcriptional control, many transcription regulatory factors were chosen, hence, genetic engineering for introgression of such genes that are known to be involved in stress response and putative tolerance, might prove to be a faster track towards improving crop varieties for enhanced cold tolerance. 

Low-temperature limitations have been overcome by the identification of cold-tolerant genes for applications in genetically transformed crops. In transgenic tobacco (*Nicotiana* *tabacum*), chilling tolerance at 1 ºC for 7 d was achieved by the over expression of a gene encoding chloroplast *w*3 fatty acid desaturase [65]. Furthermore, tolerance at 1 ºC for 11 d was conferred using a gene encoding a non-specific cyanobacterial desaturase, and the resultant transgenic tobacco plants showed a reduction in saturated fatty acid content in membrane lipids [66]. The over expression of glycerol-3-phosphate acyl transferase altered the unsaturation of fatty acids and conferred chilling tolerance in transgenic plants [67-69]. Hence, modifications in lipid composition that stabilize cell membranes and prevent cellular leakage lead to cold tolerance.

Transgenic technology has opened up many exciting possibilities to improve cold stress in plants by introduction or removal of gene or genes that regulate a specific trait [70]. It also offers uncommon opportunities for improvement in genetic potential of plants in the form of development of specific crop varieties that are more resistant to biotic and cold stresses with enhanced nutritional level. 

During last two decades, advancement in plant biotechnology has led to the identification and isolation of a number of transcription factor(s) related to cold stress tolerance. A good number of genes which have been identified in different studies (Table **1**) raise the question of exactly which genes are most central to increasing cold/ freezing tolerance. The genes selected for transformation should be involved in encoding enzymes that are required for the biosynthesis of various osmoprotectants. Other classes of genes that selected for transformation include those that encoded enzymes for modifying membrane lipids, LEA protein, and detoxification enzymes. In these studies, either a single gene for a protective protein or an enzyme was overexpressed under the control of the constitutive 35S cauliflower mosaic virus (*CaMV*) promoter in transgenic plants, although several genes have been shown to function in environmental stress tolerance and response [56]. The genes encoding protein factors that regulate gene expression and signal transduction, that function in stress responses may be useful for improving the cold tolerance of plants by gene transfer as they can regulate many stress-inducible genes involved in cold stress tolerance. 

The CBF genes represent one of the most significant discoveries in the field of low temperature adaptation and signal transduction. All important crops and few vegetables species have contained this gene [11]. Various low temperature-inducible genes have been isolated from plants that appear to be involved in tolerance to cold stress [71, 72], and the expression of some of them is regulated by C-repeat binding factor/dehydration-responsive element binding (*CBF/ DREB*1) transcription factors. Three *CBF/DREB*1 genes (*CBF3/DREB1a*, *CBF1/DREB1b*, and *CBF2/DREB1c*) belonging to the *AP*2/*DREBP* family of DNA-binding proteins have been identified in *Arabidopsis *[73, 74, 75]. Transgenic *Arabidopsis* plants constitutively over-expressing a cold inducible transcription factor (*CBF*1; *CRT/DRE* binding protein) showed tolerance to freezing without any negative effect on the development and growth characteristics [76]. Overexpression of *Arabidopsis CBF*1 (*CRT/DRE* binding protein) has been shown to activate cor homologous genes at non-acclimating temperatures [77]. The *CBF*1 cDNA when introduced into tomato (*Solanum lycopersicum*) under the control of a CaMV35S promoter improved tolerance to chilling, drought and salt stress but exhibited dwarf phenotype and reduction in fruit set and seed number per fruit [11]. 

The expression of related cold shock proteins (CSPs) from bacteria, CspA from *Escherichia coli* and CspB from *Bacillus subtilis*, promotes stress adaptation in multiple plant species [78]. Transgenic rice plants expressing CspA and CspB manifest improved stress tolerance for a number of abiotic stresses, including cold, heat, and water deficits. It has long been established that changes in gene expression occur upon exposure to cold acclimation. Number of COR genes isolated from *Arabidopsis* that encode polypeptides thought to have protective roles against dehydration. Expression profile experiments in *Arabidopsis *demonstrated that extensive changes in gene expression occur during cold acclimation and that a substantial number of the genes that are up-regulated by the cold response are involved in metabolism. Hajela *et al*. [79] reported that a set of genes that encode a related family of cold-regulated (*COR*) proteins, which are massively induced during cold acclimation [80]. These *COR* genes were used by different researchers to identify a family of *Arabidopsis* transcription factors known as either C-repeat binding factors (*CBF*) (*CBF1, CBF2 and CBF3*) or dehydration responsive element-binding factors (*DREB*) (*DREB1B, DREB1C* and *DREB1A*). In Arabidopsis (*A. thaliana*) and rice, the CBF/DREB1-dependent cold response pathway has been shown to play a predominant role in freezing tolerance through the process of cold acclimation. These *CBFs/DREBs* are upstream transcription factors that bind to promotor *cis* element *CRT/DRE* and activate the expression of these cold responsive genes [71]. Overexpression of *CBF1/DREB1b *and *CBF3/DREB1a *enhances cold tolerance by inducing *COR *(cold regulated) genes [81, 76, 75, 5]. Furthermore, its overexpression causes many biochemical changes, such as the accumulation of sugar and proline [5]. Thus, the *CBF/DREB1 *genes are thought to be activators that integrate several components of the cold acclimation response by which plants increase their tolerance to low temperatures after exposure to nonfreezing conditions [5]. Several stress induced *COR* genes such as *rd*29A, *COR*15A, *kin*1 and *COR*6.6 are triggered in response to cold treatment, ABA and water deficit stress [4]. 

There have been numerous efforts in enhancing tolerance towards multiple stresses such as cold, drought and salt stress in crops other than the model plants like *Arabidopsis*, tobacco and alfalfa. An increased tolerance to freezing and drought in *Arabidopsis* was achieved by overexpressing *CBF*4, a close *CBF*/ *DREB*1 homolog whose expression is rapidly induced during drought stress and by ABA treatment, but not by cold [82]. Subsequently, the overexpression of *DREB*1A has been shown to improve the drought- and low-temperature stress tolerance in tobacco, wheat and groundnut [83, 84]. The use of stress inducible *rd*29A promoter minimized the negative effects on plant growth in these crop species. Further, Gilmour *et al*. [80] have shown that ectopic transgenic overexpression of *CBF1/DREB1B*, *CBF2/ DREB1C* or *CBF3/DREB1A* in *Arabidopsis* activates a suite of *CBF/DREB* target genes at warm temperatures and results in increased freezing and drought tolerance. *CBF* transcripts begin accumulating within 15 minutes of plants being exposed to low temperature strongly suggests that the low temperature “Thermometer” and “Signal Transducer” are present at warm non-inducing temperatures. Two cDNA clones that encode DRE-binding proteins, *DREB1A/CBF3* and *DREB2A*, have been isolated by using yeast one-hybrid screening. cDNA clones encoding two *DREB1A* homologs (named *DREB1B/CBF1* and *DREB1C/CBF2*) and one *DREB2A *homolog (*DREB2B*) were also isolated and Expression of the *DREB1A* gene and its two homologs was induced by low-temperature stress, whereas expression of the *DREB2A* gene and its homolog was induced by dehydration. These results indicate that two independent families of DREB proteins, *DREB1* and *DREB2*, function as transacting factors in two separate signal transduction pathways under low-temperature and dehydration conditions, respectively. 

However, Agarwal *et al*. [85] showed that *CBF*s are negatively regulated by an upstream transcription factor, *MYB15* (an *R2R3-MYB* family protein) in *Arabidopsis*. *MYB15* is expressed even in the absence of cold stress, and *MYB15* can bind to *MYB* recognition elements (*MYBRS*) in the promoters of *CBFs*. *MYB15 *mutant plants show enhanced expression of CBFs during cold acclimation and enhanced freezing tolerance, whereas, transgenic *Arabidopsis* overexpressing *MYB15 *showed a decreased expression of *CBFs* and a reduction in freezing tolerance. Thus, *MYB15* is an upstream transcription factor that negatively regulates the expression of *CBFs.*

Transcriptome analysis of *ZAT12*-overexpressing *Arabidopsis* revealed that the *ZAT12* regulon consists of at least 24 cold standard set (*COS*) genes, of which nine are cold-induced and 15 are cold-repressed genes [55]. Constitutive overexpression of *Arabidopsis* *DREB1A* improved drought and low-temperature stress tolerance in tobacco, and regulation of transgene expression *via *the stress-inducible *RD29A* promoter minimized the negative effects on plant growth [83]. Similarly, the *Arabidopsis* *DREB1A* gene was placed under control of the *RD29A* promoter and transferred *via *biolistic transformation into bread wheat [84]. However, constitutive overexpression of the *CBF* genes using the cauliflower mosaic virus 35S promoter can result in undesirable agronomic traits. In *Arabidopsis*, *CBF* overexpression can cause a ‘‘stunted’’ growth phenotype, a decrease in seed yield and a delay in flowering [81, 5].

In the last decade, extensive research efforts have been undertaken to identify and characterize cold-responsive (*COR*) genes and a number of homologous components of the *Arabidopsis CBF* cold response pathway in many plants have been found [6]. Many of these putative orthologs have been structured, analyzed and functionally tested. The expression patterns of the *CBFs* and *CORs* in response to low temperature are similar in a variety of plants species, involving rapid cold-induced expression of the *CBF*s followed by expression of *CBF*-targeted genes that increase freezing tolerance. Moreover, constitutive overexpression of the *Arabidopsis* *CBF* genes in other plants resulted in increased freezing tolerance that have been successfully used to engineer cold stress tolerance in several crop species [6].

Transgenic attempts with other structural genes have also been made with fair degree of success. Genetically engineered tobacco plants over-expressing chloroplast glycerol-3-phosphate acyltransferase (*GPAT*) gene (involved in phosphatidyl glycerol fatty acid desaturation) from squash (*Cucurbita maxima*) and *A. thaliana* [86] showed an increase in the number of unsaturated fatty acids present in the plant cell wall, which enhance the cold tolerance to the plants during cold stress. Expression of a plant phosphatase (*At PP2CA*) in transgenic *A. thaliana* can accelerate the development of cold acclimation and increase freezing tolerance. It has also shown that transgenic plants expressing a constitutively active kinase *NPK*1, is more tolerant to chilling and other abiotic stresses [87]. Pennycooke *et al*. [88] reported that down-regulating α-Gal (α -Galactosidase) in transgenic petunia resulted in an increase in freezing tolerance suggesting that engineering raffinose metabolism by transformation with α -Gal provides an additional method for improving the freezing tolerance of plants. The overexpression of genes encoding *LEA* proteins can improve the stress tolerance of transgenic plants. Expression of the citrus gene encoding a *LEA* protein, *CuCOR19* increased the cold tolerance of transgenic tobacco [89]. Likewise, the freezing tolerance of *Arabidopsis* was increased by the ectopic expression of the wheat gene *WCS19* [47], the *Arabidopsis* gene *COR15A *[64], and the co-expression of the genes *RAB18 *and *COR47* and *XERO2* and *ERD10* [90]. The freezing tolerance of strawberry leaves was enhanced by expression of the wheat dehydrin gene *WCOR410* [72]. On the other hand, the expression of two cold-induced *LEA* proteins from spinach (Kaye *et al*., 1998) [91] and three desiccation-induced *LEA* proteins from *C. plantagineum* [92] in tobacco did not induce any significant changes in the freezing or drought tolerance of the respective transgenic plants. This may indicate either that not all *LEA* proteins make a significant contribution to plant stress tolerance, or that they need a particular background to function in, as suggested for transgenic strawberry plants [72]. Kim *et al*. [44] engineered tobacco with ring zinc finger protein (*RDCPt*) from hot pepper (*Capsicum annuum*) and reported that expression of this gene resulted in improved cold tolerance in transgenic plants as compared to wild type. Dai *et al*. [93] reported that overexpression of *OsMYB3R*-2 in transgenic *Arabidopsis *increased tolerance to freezing when exposed to -8 ºC for 10 h. They found that survival after 6 d at normal conditions was 26.8% for the wild-type and 84.5% for transgenic lines. Phenotypically, most transgenic seedlings were green and could regrow as compared with the wild type; whereas most wild-type seedlings became white and did not regrow after removed to normal conditions. The survival percentage under different low temperatures also showed dramatic difference between the transgenic plants and wild-type plants. 

Pramanik and Imai [94] reported that *TPP* (trehalose-6-phosphate phosphatase) genes expressed in rice and their expression is induced by cold. Trehalose accumulates rapidly and transiently, which follows the transient induction of *TPP* activity, in rice tissues during chilling stress [94]. Overexpression of *TPS* (*trehalose-6-phosphate synthase*) and *TPP* genes enhanced the accumulation of trehalose and tolerance to cold stress in transgenic tobacco and rice [95-98]. However, the regulatory mechanism of *TPPs* by cold or other stresses is unclear. In another study, Su *et al.* [99] observed that *MYBS*3 plays a critical role in cold adaptation in rice and necessary for enhancing cold tolerance. They reported that transgenic rice constitutively overexpressing *MYBS*3 tolerated 4 ºC for at least 1 week and exhibited no yield penalty in normal field conditions.

Based on previous studies it has been established that the *CBF* cold responsive pathway is an integral component of the cold acclimation response [100, 63, 6]. However, the transcriptome data showed that additional cold-regulatory pathways also exist [52, 53, 101]. Transcriptome comparisons indicated that only 12% of the cold-responsive genes are certain members of the *CBF *regulon. Moreover, at least 28% of the cold-responsive genes were not regulated by the *CBF* transcription factors, including 15 encoding known or putative transcription factors, indicating that these cold-responsive genes are members of different low-temperature regulons [55].

When overexpressed in *Arabidopsis* and tobacco, the soybean gene *SCOF-1 *(encodes a zinc-finger protein) can activate *COR *gene expression and increase freezing tolerance in non-acclimated transgenic plants, although the *SCOF-1* protein does not directly bind to either the *DRE/CRT* or the ABRE elements [102]. *SCOF-1* interacts with another G-box binding* bZIP* protein, *SGBF-1*. *SGBF-1* can activate ABRE-driven reporter gene expression in *Arabidopsis* leaf protoplasts. Thus, *SCOF-1* may regulate the activity of SGBF-1 as a transcription factor in inducing *COR *gene expression [102].

Forward genetic analysis in *Arabidopsis* identified two transcription factors, high expression of osmotically responsive genes, *HOS9* and *HOS10*, which are required for basal freezing tolerance [103, 104]. Similarly, microarray analysis led to the identification of the cold-stress-inducible *AP2* family transcription factor gene related to *ABI3/VP1* (*RAV1*) [52, 55] that might regulate plant growth under cold stress. *RAV1* is down regulated by epibrassinolide and transgenic *Arabidopsis* overexpressing *RAV1* exhibits a retardation of lateral root and rosette-leaf development. Further, the importance of *CBF*-independent pathways is also supported by analysis of mutants that have increased freezing tolerance, for example, mutations in eskimo1 (*ESK1*), a protein of unknown function, result in constitutive freezing tolerance. The *Eskimo1 *mutation was first identified as a mutation conferring frost survival without an acclimation period [105]. Later on, Bouchabke-Coussa *et al*. [106] clearly showed that *ESKIMO1 *mutants are more tolerant to freezing but only after acclimation. The genes that are affected by the *ESK1* mutation are distinct from those of the *CBF* regulon [107]. Recently, a *CBF* 1 gene has been introduced into tomato that resulted in transgenic plants showing higher activity of superoxide dismutase (SOD), higher non-photochemical quenching (NPQ), and lower malondialdehyde (MDA) content . Thus suggesting that *CBF1* protein plays an important role in protection of PSII and PSI during low temperature stress at low irradiance [108].

Aforementioned studies clearly demonstrate the complex and interactive relationships among different pathways regulated by cold acclimation. Hence, elucidation of the mechanism regulating cold-regulated genes is an important goal in achieving a full understanding of cold acclimation. With the advent of molecular genetics and biotechnology, it is now possible to genetically engineer plants to be more tolerant to low temperature. The technologies can be so developed/ modified that will significantly improve breeding efficiency, resulting in rapid and accurate incorporation of cold tolerant genes into crop plants. 

## PROSPECTS OF COLD TOLERANT GENES IN HILL AGRICULTURE

Cold is an environmental factor that limits the geographical distribution and growing season of many plant species, and it often adversely affects crop quality and productivity [71]. Most temperate plants can acquire tolerance to freezing temperature by a prior exposure to low non-freezing temperature, a process known as cold acclimatization [39,109, 110]. Plants of tropical and subtropical origins are sensitive to chilling temperature (0^o^C-10^o^C) and are incapable of cold acclimation. Genes induced by stress can be roughly classified into two groups: genes coding for regulatory proteins, mainly transcription factors, and genes encoding proteins involved directly in response mechanisms; genes from both classes are of interest. Variations in the expression of regulators could lead to a protective status before the emergence of stress and have multiple effects. Genes involved in protection or repair mechanisms could be new targets for the improvement of plant plasticity and adaptive responses to stress [111]. The unraveling of general stress responses in the model species *Arabidopsis thaliana *helped to identify potential targets for plant breeding. *Arabidopsis* genes involved in tolerance to abiotic stress were transferred, by genetic engineering, to many crops and tolerance was successfully conferred in the field, despite the complexity of plant responses to environmental stress [11, 61]. Thus, finding new key genes responsible for abiotic stress tolerance phenotypes is of great importance not only for a better understanding of stress responses, but also for promising future crop improvement.

Many studies have suggested that cold regulated gene expression is critical in plants for both chilling tolerance [47, 11] and cold acclimation [12, 71, 13, 112]. Cold responsive genes encode a diverse array of proteins such as enzymes involved in respiration and metabolism of carbohydrates, lipids, phenylpropanoids and antioxidants: molecular chaperones, antifreeze proteins, and others with a presumed function in tolerance to dehydration caused by freezing [113, 39, 71]). The change in the gene expression occur in plant during cold acclimatization a developmental process that results in increased tolerance [28]. Since then, it has repeatedly been speculated that certain *COR* (cold regulated) genes might have role in freezing tolerance. To test this notion investigators have turned to isolating the characterizing genes that are expressed in response to low temperature. These efforts have led to the identification of a number of genes such as the *COR 15a KINI, LTI 78, fad 7*etc. of *A. thaliana*. The generic trends of the genes and transcription factors are available in STIFDB (Stress responsive Transcription Factor Database). STIFDB (available at http://caps.ncbs.res.in/stifdb.) is a database of stress-related genes, which are upregulated in abiotic stress-related microarray experiments. STIFDB provides a platform to understand the stress-regulome of abiotic stress responsive genes in plants. STIFDB will be a highly useful resource for a researcher working on abiotic stress responses in plants [114].

Low temperature stress is a major environmental factor that not only limits where crops can be grown but also reduces yields depending on the weather in a particular growing season. In addition to exceptionally stressful years that cause significant yield reductions, less extreme stress almost certainly causes smaller losses over large areas to produce comparable yield reductions every year. Even in cases when freezing stress does not result in yield loses; it often results in crop quality reduction. Each year, worldwide losses in crop production due to low temperature damage amount to approximately $ 2 Billion. Some of the major losses include the 1995 early fall frosts in the US which caused losses of over $1 billion to corn and soybeans. The occasional freezes in Florida have shifted the citrus belt further south, and California sustained $650M of damage in 1998 to the citrus crop due to a winter freeze. The inherent cold hardiness of the crop determines in which agricultural areas it can be grown. Crops that are more resistant to freezing stress would allow some geographical regions to grow more profitable and productive crops with less environmental risks. However despite continued efforts, traditional breeding has had only limited success in imparting crop plants with better freezing tolerance due to very little was known about the mechanisms that regulate chilling and freezing tolerance. With the advent of molecular genetics and biotechnology, it is now possible to genetically engineer plants to be more tolerant to many environmental adversities, including low temperature. Molecular studies have shown that several genes with various functions are induced by environmental stresses such as drought, high-salinity and low temperature in plants. Most of the dehydration responsive genes are induced by the plant hormone abscisic acid (ABA), but others are not. Expression analyses of dehydration-responsive genes have provided at least four independent regulatory systems (regulons) for gene expression in a model plant *Arabidopsis thaliana*. The *cis*-acting elements in the promoters of some genes that have a typical stress-inducible expression profile and the transcription factors that affect the expression of these genes have been analyzed [115]. 

## FUTURE OUTLOOK AND CONCLUSION

The development of genetically engineered plants by the introduction and/or overexpression of selected genes seems to be a viable option to hasten the breeding of ‘‘improved’’ plants. Intuitively, genetic engineering would be a faster way to insert beneficial genes than through conventional or molecular breeding. Also, it would be the only option when genes of interest originate from cross barrier species, distant relatives, or from non-plant sources. Applications of genomic approaches and gene knockout strategies are progressing to accelerate efforts to assess systematically and understand complex quantitative traits such as acquired tolerance to temperature extremes. By using genetic and molecular approaches, a number of relevant genes have been identified and new information continually emerges to enrich the *CBF* cold-responsive pathway. Thus, the *CBF/DREB*1 genes are thought to be activators that integrate several components of the cold acclimation response by which plants increase their tolerance to low temperatures after exposure to non-freezing conditions. The *DREB1/CBF *genes have been successfully used to improve abiotic stress tolerance in a number of different crop plants. Studies on the other transcription factors associated with stress response are in progress.

However, the results of the transcriptome study demonstrate the highly complex nature of plant adaptation to low temperature. To overcome this problem a transgenic approach to promoting cold tolerance has been widely adopted, with some success. For example, increasing the accumulation of two compatible solutes, that is, glycinebetaine and trehalose, in transgenic rice by overexpressing either *E*. *coli *choline oxidase, or trehalose-6-phosphate synthase fused to trehalose-6- phosphate phosphatase, enhanced tolerance to both salt and cold. In fact a large number of genes identified in different studies have currently annotated with ‘‘unknown function’’ and involve new genes and new pathways indicates that our knowledge of the transcriptional control of the low temperature response is limited, and the regulation of these transcriptional responses is far more complex than previously believed. Information on the low-temperature transcriptome, proteome and metabolome is expected to continue to increase in the near future. This information is necessary for our understanding of the complex network of molecular changes that are important for chilling and freezing tolerance in crop plants. A well focused approach combining the molecular, physiological and metabolic aspects of cold stress tolerance is required for bridging the knowledge gaps between short- and long-term effects of the genes and their products, and between the molecular or cellular expression of the genes and the whole plant phenotype under stress. Collaborative research with many research groups to improve stress tolerant crop plants utilizing regulon biotechnology were undertaken under the aegis of CGAIR. We hope the results of these collaborative studies will contribute to the sustainable food production in developing countries and help to prevent the global-scale environmental damage.

## Figures and Tables

**Table 1:** Selective Reports on Production of Cold Stress-Tolerant Transgenic Crops

Gene (s) / Gene product	Cellular role	Transgenic Host-Plant	Performance of transgenic plants	Reference
*gpat * Glycerol 3-phosphate acyltransferase	Fatty acidunsaturation	*N*. *tabacum*	Transformants showed less chilling damage to photosynthetic activity than the wild type	[86]
*sod * Superoxide dismutase	Dismutation of toxic reactive oxygen intermediate	*N*. *tabacum*	Transformants showed 20% higher photosynthetic activity during chilling compared to untransformed plants	[116]
*sacB * Levan sucrase	Fructan biosynthesis	*N*.*tabacum*	Transformants were more tolerant to freezing and *PEG*-mediated water stress than the wild type	[117]
*cor15a* Cold regulated gene	Promotes freezing tolerance	*A. thaliana*	Transformants showed *in vivo* enhanced freezing tolerance of protoplasts and the chloroplasts	[64]
*mn-sod * Mn-Superoxide dismutase	Dismutation of reactiveoxygen inter mediates in mitochondria	*M*. *sativa*	Transformants showed reduced injury from water deficit stress and increased winter survival	[118]
*gst*/*gpx * Glutathione-Stransferase and glutathione peroxidase	Detoxification of herbicides and toxic substances	*N*.*tabacum*	Transformants over-expressing *GST/GPX* showed stimulated seedling growth under chilling and salt stress	[119]
*cbf1 * CRT/DRE binding factor	Transcription factor	*A. thaliana*	Transformants showed regulation of several *cor *genes at the same time and showed freezing tolerance	[76]
*dreb1 anddreb2* DRE-binding Protein	Transcription factor	*A. thaliana*	Transformants revealed freezing and dehydration tolerance but caused dwarfed phenotypes in transgenic plants	[81]
*WCS120/COR39 * CCGAC sequences like CRT/DREs in its promoter	Low temperature regulated gene	*Trtricum sativum*	cold inducible in monocotyledonous and dicotyledonous plants	[120]
*codA * Choline oxidase A	Glycinebetaine biosynthesis	*O*. *sativa*	Transformants accumulated high levels of glycinebetaine and showed increased tolerance to salt and low temperature stress	[121]
*codA * Choline oxidase A	Glycinebetaine biosynthesis	*A*. *thaliana*	Transformants were tolerant to salt and cold	[122]
*DREB1A (CBF3)* DRE-binding protein	Transcription factor	*Arabidopsis*	Increased salt, drought and cold tolerance in nonacclimated plants	[75]
*prodh * Proline dehydrogenase	Proline biosynthesis	*A*. *thaliana*	The antisense transgenics were more tolerant to freezing and high salinity than wild types	[123]
*CBF3* DRE-binding protein	Transcription factor	*Arabidopsis*	Increased freezing tolerance of cold- acclimated plants	[5]
*ala1 * Aminophospholipid ATPase 1	P-type ATPase (Transporter protein)	*A*. *thaliana*	Transformants showing down regulation results in cold-affected plants that are much smaller than the wild type	[34]
*SCOF1*cold-inducible zinc finger protein	Regulator of *SGBF-1* as a transcription factor	*Glycine max*	activate *COR *gene expression and increase freezing tolerance in non-acclimated transgenic plants	[102]
*abi3 *Abscisic acid induced protein	Transcription factor	*A. thaliana*	Marked increase in expression of low temperature-induced freezing tolerance accompanied by up-regulation of *RAB18, LTI129, LTI130 and LTI178*	[13]
*CuCOR19*citrus dehydrin	Inhibition of lipid peroxidation	*N*. *tabacum*	Increased the cold tolerance	[89]
*CBF1/ DREB1b*DRE-binding protein	Transcription factor	*O*. *sativa*	The cold-responsive genes lip5, lip9, and OsDhn1 were up-regulated in the transgenic plants	[124]
*DREB1A* (*rd29A)* DRE-binding protein	Stress-inducible promoter	*N*. *tabacum*	Improved drought and low-temperature stress tolerance	[83]
*OSISAP1* Zinc-finger protein	Transcription factor	*N*. *tabacum*	The transcript level of *OSISAP*1 was increased to a very high level during a 12-h cold treatment	[125]
*Osmyb4*	Transcription factor	*Arabidopsis*	Increases chilling and freezing tolerance	[126]
*HOS10* Encodes an R2R3-type protein	Transcription factor	*O*. *sativa*	Enhanced cold tolerance	[127]
*ZAT12* C2H2 zinc finger	Transcription factor	*Arabidopsis*	Improved cold acclimation	[55]
*Cor15am*Chloroplast stromal protein	Stress-inducible promoter	*Arabidopsis*	Enhanced cryoprotective activity	[128]
*OsMYB3R-2* DNA-binding domain	Transcription factor	*Arabidopsis *	Overexpression of *OsMYB3R-2* leads to increased tolerance to freezing, drought, and salt stress	[93]
*ACBP6*Acyl-CoA-binding protein	Decline in phosphatidylcholine and elevation of phosphatidic acid	*Arabidopsis*	Overexpression of *ACBP6 *enhanches freezing tolerance	[129]
*OsMYB3R-2* DNA-binding domain	Transcription factor	*O*. *sativa*	Overexpression of OsMYB3R-2 exhibited enhanced cold tolerance	[130]
AtCSP3 Cold shock protein	RNA chaperon	*Arabidopsis*	Transgenic plants conferred enhanced freezing tolerance as compared to wild type plants hence demonstrating essential role of RNA chaperones for cold adaptation in higher plants	[131]
*MYBS3* DNA-binding repeat MYB	Transcription factor	*O*. *sativa*	Plays a critical role in cold adaptation in rice	[99]
*mybc1*Regulate osmotic stress tolerance	Transcription factor	*Arabidopsis*	Exhibited an increased tolerance to freezing stress	[132]
*ThpI* Thermal hysteresis proteins (Anti freeze protein)	Transcription factor	*Arabidopsis*	Enhanced low temperature tolerance in transgenic plants was observed by changes of electrolyte leakage activity, malonyldialdehyde and proline contents	[133]
*CBF1* CRT/DRE binding factor 1	Transcription factor	*Solanum Lycopersicum*	Detection of higher activity of superoxide dismutase (SOD), higher non-photochemical quenching (NPQ), and lower malondialdehyde (MDA) content in transgenic tomato leaves suggest that CBF1 protein plays an important role in protection of PSII and PSI during low temperature stress at low irradiance	[108]
